# SALL4 Expression in Gonocytes and Spermatogonial Clones of Postnatal Mouse Testes

**DOI:** 10.1371/journal.pone.0053976

**Published:** 2013-01-11

**Authors:** Kathrin Gassei, Kyle E. Orwig

**Affiliations:** Department of Obstetrics, Gynecology & Reproductive Sciences, Magee-Womens Research Institute, University of Pittsburgh School of Medicine, Pittsburgh, Pennsylvania, United States of America; National Cancer Institute, United States of America

## Abstract

The spermatogenic lineage is established after birth when gonocytes migrate to the basement membrane of seminiferous tubules and give rise to spermatogonial stem cells (SSC). In adults, SSCs reside within the population of undifferentiated spermatogonia (A_undiff_) that expands clonally from single cells (A_single_) to form pairs (A_paired_) and chains of 4, 8 and 16 A_aligned_ spermatogonia. Although stem cell activity is thought to reside in the population of A_single_ spermatogonia, new research suggests that clone size alone does not define the stem cell pool. The mechanisms that regulate self-renewal and differentiation fate decisions are poorly understood due to limited availability of experimental tools that distinguish the products of those fate decisions. The pluripotency factor SALL4 (sal-like protein 4) is implicated in stem cell maintenance and patterning in many organs during embryonic development, but expression becomes restricted to the gonads after birth. We analyzed the expression of SALL4 in the mouse testis during the first weeks after birth and in adult seminiferous tubules. In newborn mice, the isoform SALL4B is expressed in quiescent gonocytes at postnatal day 0 (PND0) and SALL4A is upregulated at PND7 when gonocytes have colonized the basement membrane and given rise to spermatogonia. During steady-state spermatogenesis in adult testes, SALL4 expression overlapped substantially with PLZF and LIN28 in A_single_, A_paired_ and A_aligned_ spermatogonia and therefore appears to be a marker of undifferentiated spermatogonia in mice. In contrast, co-expression of SALL4 with GFRα1 and cKIT identified distinct subpopulations of A_undiff_ in all clone sizes that might provide clues about SSC regulation. Collectively, these results indicate that 1) SALL4 isoforms are differentially expressed at the initiation of spermatogenesis, 2) SALL4 is expressed in undifferentiated spermatogonia in adult testes and 3) SALL4 co-staining with GFRα1 and cKIT reveals distinct subpopulations of A_undiff_ spermatogonia that merit further investigation.

## Introduction

Spermatogonial stem cells (SSC) are located on the basement membrane of the seminiferous tubules and drive spermatogenesis throughout adult life by balancing self-renewal and differentiation cell divisions. SSCs are generally considered to be among the cohort of undifferentiated type A spermatogonia (A_undiff_), the most primitive cell type in the testis (for review see [1]). A_undiff_ are arranged as single cells (A_single_ or A_s_), pairs of cells (A_paired_ or A_pr_) and chains of four to 16 or 32 cells (A_aligned_ or A_al_) [2]. Among the undifferentiated spermatogonia, the A_s_ spermatogonia and some A_pr_ spermatogonia are the most likely candidates to contain stem cell activity, though limited stem cell potential of larger clones has been speculated [3]. It has been estimated that in the mouse, A_s_ spermatogonia represent only 0.03% of germ cells in the testes [4]. The pool of adult SSCs is established after birth by the transient population of gonocytes. This cell population is quiescent after birth, but re-enters the cell cycle around postnatal day 3. The gonocytes then migrate to the seminiferous tubule basement membrane where some will establish the pool of SSCs and others will differentiate directly into cKIT-positive differentiating spermatogonia and give rise to the first wave of spermatogenesis. [5], [6]. This phase of germ lineage development is therefore crucial to initiate and maintain male fertility.

Here we analyzed the expression of the pluripotency factor SALL4, a member of the *sal* gene family of zinc-finger transcription factors, in the male mouse germline during postnatal development and during steady-state spermatogenesis. Members of this family are highly conserved between species; homologues have been identified in Drosophila [7], [8], Xenopus [9], zebrafish [10], chicken [11], mice [12] and humans [13]. In mice and humans, four sal proteins (SALL 1–4) have been described that are important for normal development and that are implicated in various congenital disorders [14].

During embryo development, *Sall4* regulates axis formation [15] and expression was observed in progenitor populations of brain, neural tube, heart, pituitary gland, somites and limbs, as well as in fetal hepatic stem/progenitor cells [16], [17]. Proper *Sall4* action in these organs during development is crucial, as evidenced by the mutant phenotype. Mutations in the human *SALL4* locus cause Okihiro syndrome, a rare autosomal-dominant condition characterized by limb and cardiac malformations, hearing loss, strabismus and postnatal growth retardation [18], [19]. Several *Sall4* mutant mice have been developed [16], [20], [21], [22]. Homozygous mice were embryonic lethal in these models, and haploinsufficiency mimicked the human phenotype in varying degrees, exhibiting modest to severe phenotypes and postnatal death of pups within the first weeks after birth. Although in one study the heterozygous mutants were able to produce small litters when intercrossed or backcrossed to a C57BL/6 background [20], the testicular phenotypes of the mutants have not been evaluated systematically in these studies. Conditional knock-out mice where Sall4 was deleted in embryonic germ cells failed to establish a robust stem cell pool, resulting in many agametic tubules [23]. Postnatal deletion of Sall4 in Stra8-expressing germ cells did not impair the onset of spermatogenesis, and germ cell differentiation appeared normal in young adult mice. However, increased seminiferous tubule degeneration was observed in older mice, indicating a role of SALL4 for stem cell pool homeostasis [23].

Hobbs et al. demonstrated that SALL4 can interact directly with PLZF, a transcription factor required for SSC function and proposed that the titration of these two transcription factors regulates their distribution on the chromatin and consequently target gene regulation [23]. To gain additional insights about this novel regulatory mechanism in the context of spermatogenic lineage development, we evaluated SALL4 expression in the developing testis on postnatal days 0, 3, 7 and 14, the period when gonocytes give rise the SSCs and the first round of spermatogenesis. In addition, we correlated SALL4 expression in adult testes with established markers of undifferentiated (GFRα1, PLZF, LIN28) and differentiating (cKIT) spermatogonia as well as with clone size (A_s_, A_pr_, A_al_, etc.) in whole mount preparations of seminiferous tubules.

## Materials and Methods

### Animals

C57BL/6 mice were obtained from the Jackson Laboratory (Bar Harbor, ME) and maintained in the laboratory animal housing facility at Magee-Womens Research Institute. All animal procedures were approved by the Institutional Animal Care and Use Committees of Magee-Womens Research Institute and the University of Pittsburgh. All procedures were performed in accordance with the NIH Guide for the Care and Use of Laboratory Animals (Assurance # 3654-01).

### Antibodies

The following primary antibodies were used for immunoblotting, immunofluorescence staining of testis tissue sections, or whole mount seminiferous tubules: mouse anti-ß actin (No. A1978, Sigma, St. Louis, MO); mouse anti-DAZL (ab17224, Abcam, Cambridge, MA); rabbit anti-SALL4A/B (ab29112, Abcam); mouse anti-SALL4A (ab57577, Abcam), goat anti-GATA4 (SC-1237, SantaCruz); goat anti-PLZF (AF2944, R&D Systems Inc., Minneapolis, MI); goat anti-LIN28 (AF3757, R&D Systems); goat anti-GFRα1 (AF650, R&D Systems); rabbit anti-VASA/DDX4 (ab13840, Abcam); armenian hamster anti-cKIT (H2C7, [24]).

### Protein Isolation and Western Blotting

Testes from PND 0, 3, 7 and 14 mice were dissected, the tunica albuginea removed and collected in chilled RIPA buffer (150 mM NaCl. 0.1% SDS, 1% NP-40, 50 mM Tris pH 7.5, 0.5% Sodium-Deoxycholate) for preparation of whole testis protein lysates. Tissues were disrupted mechanically and incubated on ice for 30 min and lysates were cleared from residual cell debris by centrifugation for 10 min at 10,000×g. The supernatant was collected and protein concentrations were determined by Bradford colorimetric assay (Protein Assay No.500-0006, Biorad, Hercules, CA). Protein (10 µg) was separated by SDS-PAGE on a 10% gel (Biorad Mini-PROTEAN® TGX™ Precast Gel #456-1033) and transferred onto a nitrocellulose membrane using standard techniques. Blots were blocked with 5% Blotto (No. sc-2324, Santa Cruz Biotechnology Inc., Santa Cruz, CA) in 1× Dulbecco’s Phosphate Buffered Saline (DPBS, No. 14200166, Invitrogen, Carlsbad, CA) containing 0.1% Tween-20 (P5927, Sigma) for 1 hour at room temperature. Primary antibodies rabbit anti-SALL4 (1∶1000) and mouse anti-beta actin (1∶10000) were incubated overnight at 4°C. After washing the blots three times for 10 min in DPBS-T, horseradish peroxidase-conjugated anti-rabbit IgG or anti-mouse IgG (1∶5000 dilution) were incubated for 1 hour at room temperature and protein bands were detected with SuperSignal West Pico Chemiluminescent Substrate as instructed (PI34080, Thermo Fisher Scientific, Pittsburgh, PA). ImageJ software (NIH, Bethesda, MD) was used for densitometry to evaluate relative levels of SALL4A, SALL4B and total SALL4 protein during testis development.

### Immunofluorescence Staining of Testis Tissue Sections

Testes from neonatal (postnatal day (PND) 0 and PND 3), juvenile (PND 7 and PND 14) and adult (>6 weeks of age) mice were dissected and fixed in 4% PFA for 2 hours (neonatal testes) or overnight (juvenile and adult testes) at 4°C. Fixed tissues were processed for paraffin embedding and 5 µm serial sections were collected. Sections were deparaffinized in xylene (2×15 min), rehydrated in a graded ethanol series (2×100% for 10 min, 1×95% for 5 min, 1×80% for 5 min, 1×70% for 5 min, 1×50% for 5 min, 1×25% for 5 min) and rinsed in 1× DPBS. Slides were then incubated in sodium citrate antigen retrieval buffer (10 mM Sodium Citrate, 0.05% Tween-20, pH 6) for 30 min at 97.5°C and allowed to cool to room temperature. After rinsing twice in 1× DPBS containing 0.1% Tween-20 (DBPS-T), unspecific binding sites in tissue sections were saturated by incubation with blocking buffer (1× DPBS containing 3% bovine serum albumin, 0.1% Triton X-100 and 5% normal serum from the host species of the secondary antibody) for 30 min at room temperature. Primary antibodies were diluted in blocking buffer and added to tissue sections for 90 min at room temperature. Isotype matched normal IgG at a comparable concentration was used instead of primary antibody in negative controls (see Figures S1 and S2). After washing the slides in DPBS-T 3× for 5 min, samples were incubated with the appropriate secondary antibody (goat IgG or donkey IgG) conjugated to AlexaFlour-488 or AlexaFluor-568 dye (all Molecular Probes, Invitrogen) for 45 min at room temperature. Sections were washed 3×5 min with DPBS-T and 1×5 min with DPBS before mounting with Vectashield Mounting Medium with DAPI (H-1200, Vector Laboratories, Burlingame, CA).

### Immunofluorescence Staining of Whole Mount Seminiferous Tubules

Testes from adult C57/Bl6 male mice were collected in chilled DPBS and the tunica albuginea was carefully removed. The seminiferous tubules were gently teased apart with fine forceps and briefly digested with 1 mg/ml DNase I (No. DN-25, Sigma) and 1 mg/ml Collagenase IV (No. C5138, Sigma) in DPBS. Tubules were fixed overnight in 4% PFA at 4°C and then extensively washed 3–4 times with DPBS at 60-minute intervals. For SALL4, PLZF and LIN28 staining, tubules were dehydrated in a graded methanol series (25%, 50%, 75%, 95% and 2× in 100% MeOH) for 10 min at room temperature. Samples were permeabilized for 3 hours at room temperature in a 4∶1∶1 mix of MeOH/DMSO/H_2_O_2_. Sequential rehydration was by incubation in 50% MeOH (10 min) and 25% MeOH (10 min) and DPBS (2×10 min). Samples were blocked with ice cold PBSMT blocking buffer (1×DPBS +2% blotto milk powder +0.5% Triton X-100) for 2×15 min and 1×2 hours and incubated in primary antibody at 4°C overnight. Isotype matched normal IgG at a comparable concentration was used instead of primary antibody as a negative control (see Figure S3).

Samples were washed with ice-cold PBSMT (2×15 min, 5×1 hr) and incubated in AlexaFluor-conjugated secondary antibody overnight at 4°C. Samples were washed again with ice-cold PBSMT (2×15 min, 5×1 hr) followed by DPBS (2×10 min) and mounted on raised coverslips with Vectashield mounting medium containing DAPI nuclear stain. For co-staining of SALL4 with the surface antigens GFRα1 or cKIT, tubules were only shortly dehydrated in DPBS/10% MeOH/0.1% Triton X-100 at 4°C for 60 min and blocked with PBSMT at 4°C for 60 min. Primary antibodies were diluted in PBSMT and incubated overnight at 4°C. Samples were washed 6×15 min at room temperature with DPBS/0.1% Triton X-100 (PBSTx) and incubated with donkey anti-goat Alexa568 (No. A-11057, Molecular Probes, Invitrogen) or goat anti-armenian hamster Rhodamine-Red-X (No. 127-295-160, Jackson Immuno Research, West Grove, PA) conjugates for 1 hour at 37°C. Samples were washed 6×15 min at room temperature with PBSTx and mounted on raised coverslips with Vectashield mounting medium containing DAPI.

### Imaging and Image Analysis

Sections and tubules where imaged using an Eclipse 600 fluorescent microscope (Nikon USA, Melville, NY) equipped with an X-Cite 120 fluorescence source (EXFO Life Sciences, Ontario, Canada) and a Spot RT 2.3.1 slider camera (Diagnostic Instruments, Sterling Heights, MI). A dual-emission FITC/TRITC filter was used to observe green and red fluorescent signals. Image analysis was performed using MetaVue software (Molecular Devices, Sunnyvale, CA).

Stained tissue sections from at least three adult animals were used for each co-staining experiment and a minimum of 100 circular cross sections per animal was evaluated for quantification of co-expression patterns. Whole mount tubules were mounted in small fragments of several mm in length and a minimum of 500 spermatogonial clones were scored in at least three animals for each experiment. Spermatogonial clones were defined according to Tegelenbosch and de Rooij [4]. The combined number of all single- and/or double-stained spermatogonia was used as the denominator to calculate cell ratios.

## Results

### Differential Expression of SALL4 Isoforms A and B during Postnatal Testis Development

Previous work by Wang et al. suggested that the Sall4 gene is expressed in pre-meiotic spermatogonia in the postnatal day 7 mouse testis [25]. These observations were confirmed in the recent studies of Eildermann et al. [26] and Hobbs et al. [23]. The first week after birth is critical for male germline development. During this time, gonocytes re-enter mitosis and migrate to the seminiferous tubule basement membrane where some will establish the pool of SSCs and others will differentiate directly into cKIT-positive differentiating spermatogonia and give rise to the first wave of spermatogenesis [6].

**Figure 1:**
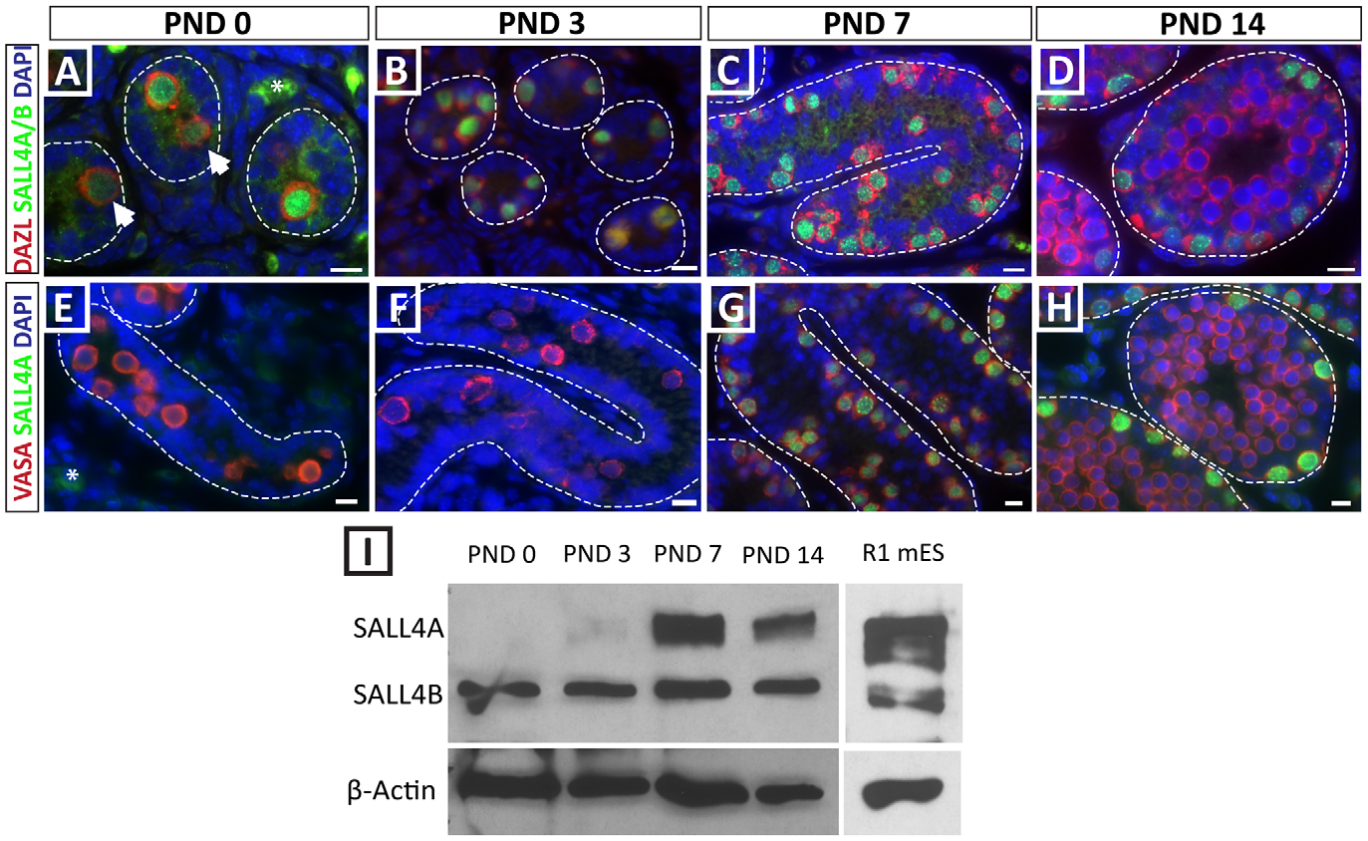
Differential expression of SALL4 isoforms during postnatal testis development. (A–D) Co-staining for DAZL (all germ cells, red) and SALL4A/B (green). (A, B) Some SALL4-negative gonocytes (white arrows) were observed at PND 0. Autofluorescence was observed in the interstitium (asterisk). All germ cells strongly express SALL4 at PND 7 (C) and PND 14 (D) (E–H) Co-staining for VASA/DDX4 (all germ cells, red) and SALL4A (green). (E,F) SALL4A was absent from VASA-positive germ cells at PND 0 and 3. SALL4A was strongly expressed in all germ cells at PND 7 (G) and PND 14 (H). Scale bars = 10 µm. (I) Western blot analysis of total testis protein isolated from different postnatal ages showed differential expression of SALL4 isoforms. Protein from R1 mouse embryonic stem cells is shown as positive control. Blots were reprobed with anti-beta actin IgG to ensure equal loading of proteins.

The mammalian Sall4 gene encodes three alternative splice variants (SALL4A, SALL4B, SALL4C). SALL4A and SALL4B were recently shown to have isoform-specific target genes and to play different roles for pluripotency maintenance and differentiation of mouse embryonic stem cells [27]. In this context we wanted to determine which of these isoforms is present during the establishment of the spermatogonial stem cell pool and the initiation of spermatogenesis in the postnatal mouse testis. Germ cells in sections from PND 0, 3, 7, and 14 were identified by staining for either DAZL or VASA, two ubiquitous germ cell markers, that were confirmed by double-immunohistochemistry to be expressed in all germ cells at the selected ages (data not shown). For co-staining experiments, we used a polyclonal antibody that recognizes both SALL4A and SALL4B in combination with anti-DAZL antibody, and we also used a monoclonal antibody that specifically recognizes SALL4A in combination with anti-VASA antibody. The data in Figure 1 indicate that while the SALL4A/B antibody stains DAZL+ germ cells on postnatal days 0, 3, 7 and 14 (Fig. 1A–D); the SALL4A-specific antibody stains VASA+ germ cells only on days 7 and 14 (Fig. 1E–H). These results were also confirmed by Western blot with the SALL4A/B antibody that distinguishes SALL4A and SALL4B isoforms based on size (Fig. 1I).

**Figure 2:**
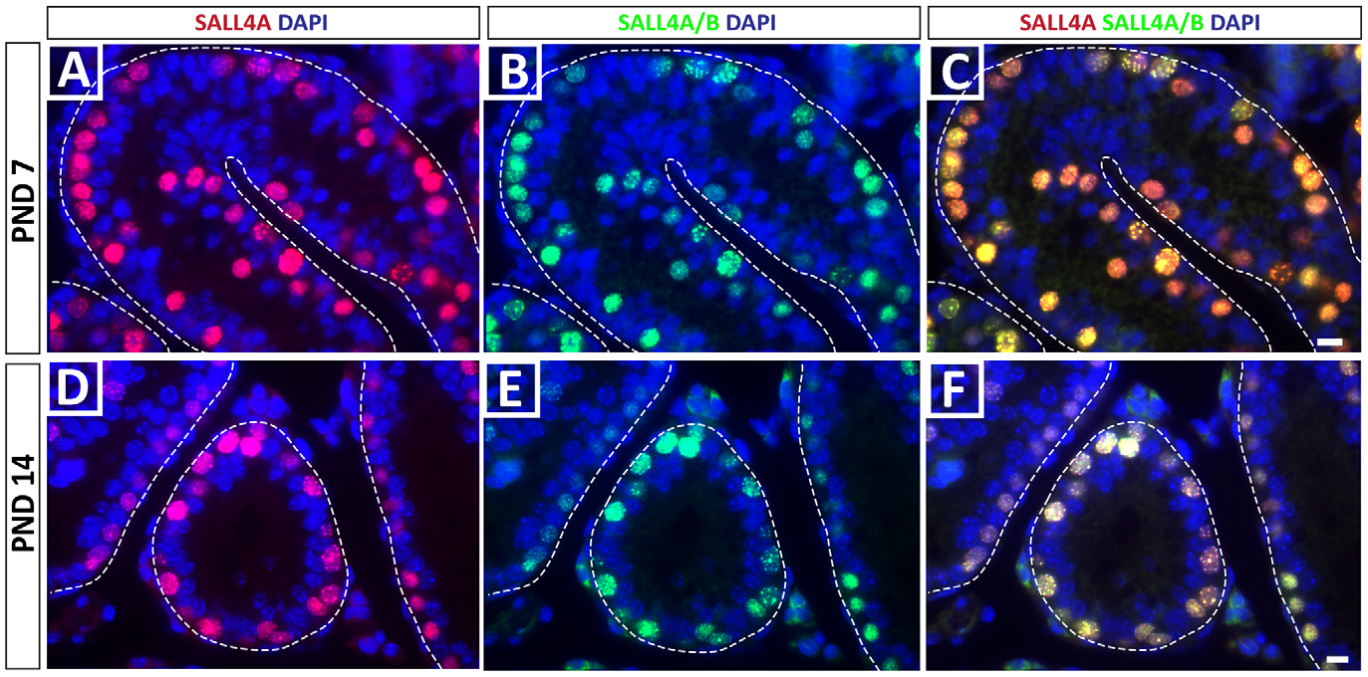
SALL4A and SALL4B co-expression in juvenile germ cells. SALL4A and SALL4B are expressed in the same population of germ cells at PND 7 (A–C) and PND 14 (D–F). Scale bars = 10 µm.

**Figure 3:**
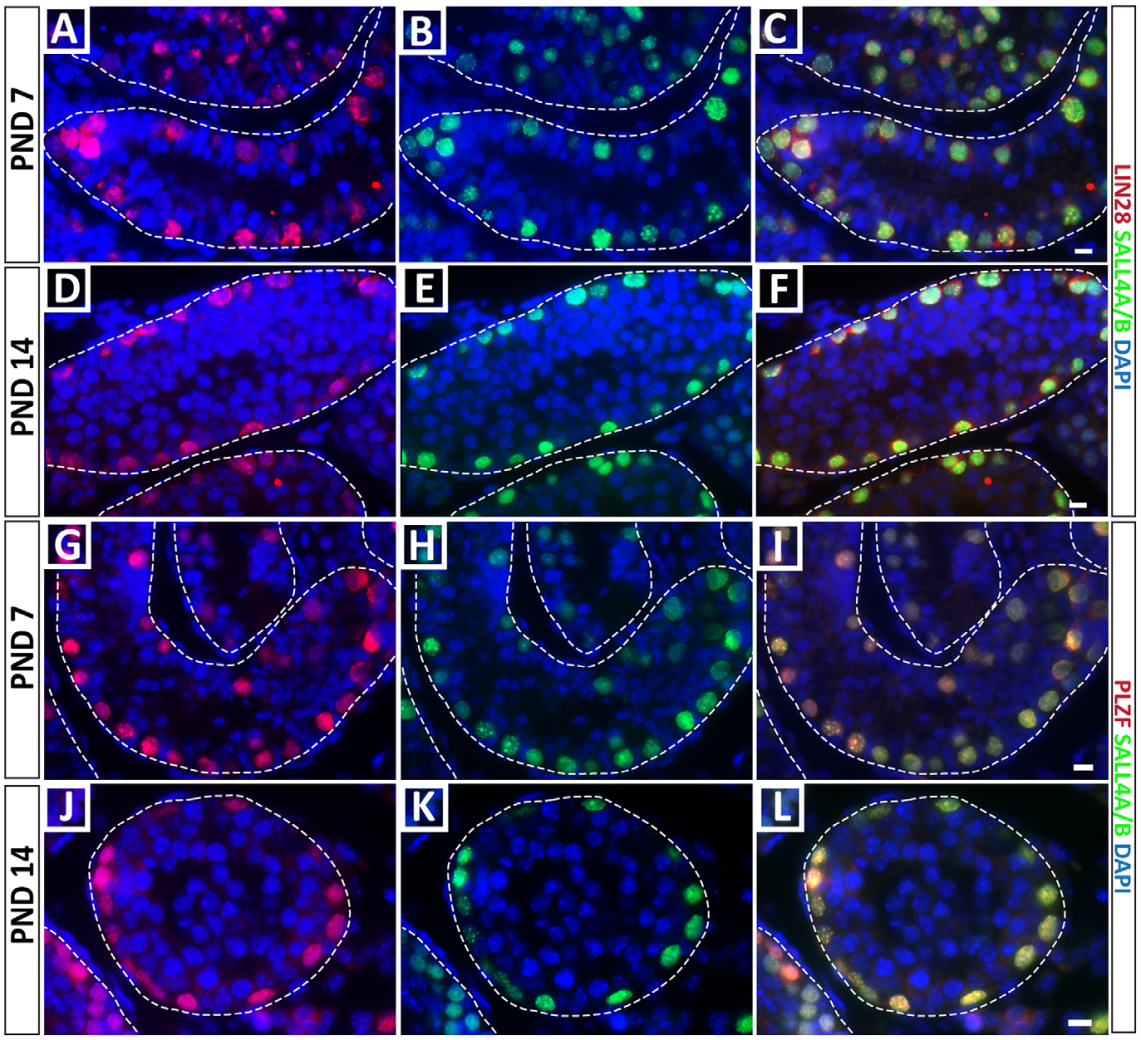
SALL4 is expressed by undifferentiated spermatogonia in postnatal day 7 and postnatal day 14 testes. (A–F) SALL4 expression is restricted to LIN28 positive undifferentiated spermatogonia at PND 7 and PND 14. (G–L) SALL4 expression is restricted to PLZF positive undifferentiated spermatogonia at PND 7 and PND 14. Scale bars = 10 µm.

We next tested the hypothesis that SALL4A and SALL4B might be expressed in different subpopulations of spermatogonia in the developing mouse testis by co-staining with the SALL4A/B and SALL4A antibodies. However, the data in Figure 2 indicate a complete overlap of staining with these two antibodies on days 7 and 14 of development, corresponding to times when the spermatogonial stem cell pool is established, and spermatogenesis is initiated. Therefore, SALL4A and B do not appear to mark different subpopulations of spermatogonia at these stages of development. To confirm the spermatogonial subtypes that express SALL4 in postnatal day 7 and 14 testes, we co-stained with the SALL4A/B antibody and LIN28 or PLZF, which are established markers of undifferentiated spermatogonia. We observed nearly complete overlap of SALL4 expression with both LIN28 and PLZF (Fig. 3). Therefore, SALL4 expression appears restricted to LIN28+ and PLZF+ undifferentiated spermatogonia at these stages of mouse testis development.

### SALL4 is Expressed in Undifferentiated Spermatogonia in the Adult Mouse Testis

**Figure 4:**
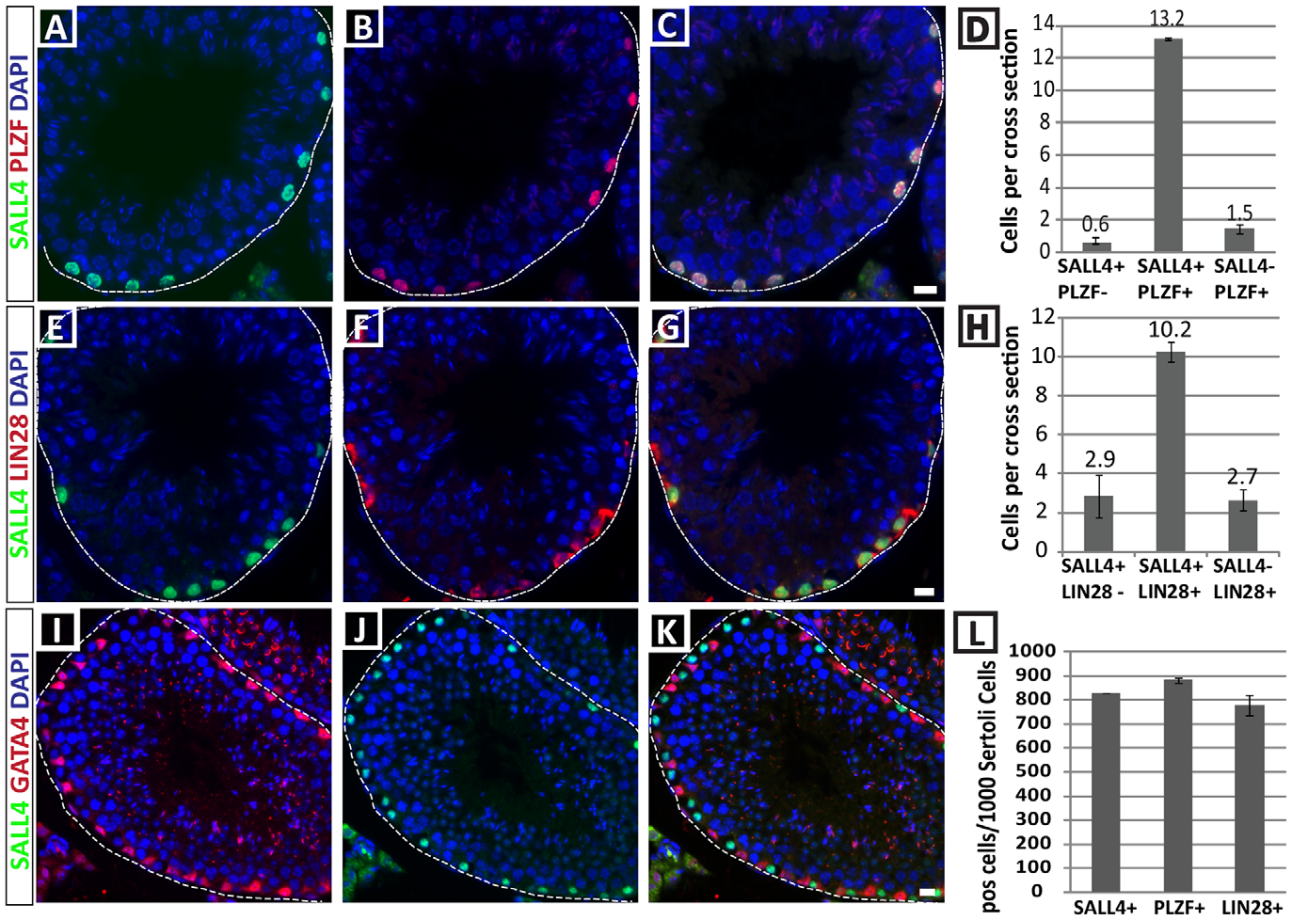
SALL4 is expressed in undifferentiated spermatogonia in adult mouse seminiferous tubules. Double immunohistochemistry of tissue sections was used to examine the localization of SALL4 in comparison to PLZF (A–D) and LIN28 (E–H), two known markers for undifferentiated spermatogonia. (D, H) Data are expressed as the average number of single or double stained cells per tubular cross section ± SEM. (I–K) SALL4 was not expressed in GATA4-positive Sertoli cells. (L) SALL4, PLZF and LIN28-positive cells were normalized to 1000 Sertoli cells to express the relative size of the respective spermatogonial populations. The basement membrane of each tubule is marked by a dashed line. Scale bars = 10 µm. For quantitative analysis, 100 circular tubule cross sections in testes from at least 3 adult male mice were scored.

To investigate the expression of SALL4 during steady-state spermatogenesis in the adult mouse testis, we co-stained tissue sections with SALL4 and the established spermatogonia markers, PLZF and LIN28, and the Sertoli cell marker GATA4 (Fig. 4). Co-expression analysis showed that SALL4 co-localizes with PLZF in 13.2 cells per tubular cross section (86.7%±1.9 overlap) and only occasionally spermatogonia were observed that expressed either SALL4 alone (0.6 cells/cross section; 3.8%±0.9) or PLZF alone (1.5 cells/cross section; 9.6%±1.3). Similarly, co-expression of SALL4 with LIN28 was found in 10.2 cells/cross section (66.3%±6.3), while 2.9 cells/cross section were SALL4 positive only (17.3%±4.7) and 2.7 cells/cross section were positive for LIN28 alone (16.5%±1.7). In contrast, SALL4 was not expressed in GATA4-positive Sertoli cells (16.6 cells/tubule cross section). Overall, 829 SALL4-positive spermatogonia, 883 PLZF-positive spermatogonia, and 778 LIN28-positive spermatogonia were observed per 1000 Sertoli cells in the adult testis based on these cell counts in adult mouse testicular sections (Fig. 4L). In summary, these data demonstrate that SALL4 is expressed by the majority of PLZF+ and LIN28+ undifferentiated spermatogonia during steady-state spermatogenesis.

### Clonal Organization of SALL4 Expressing Cells in Seminiferous Tubules

**Figure 5:**
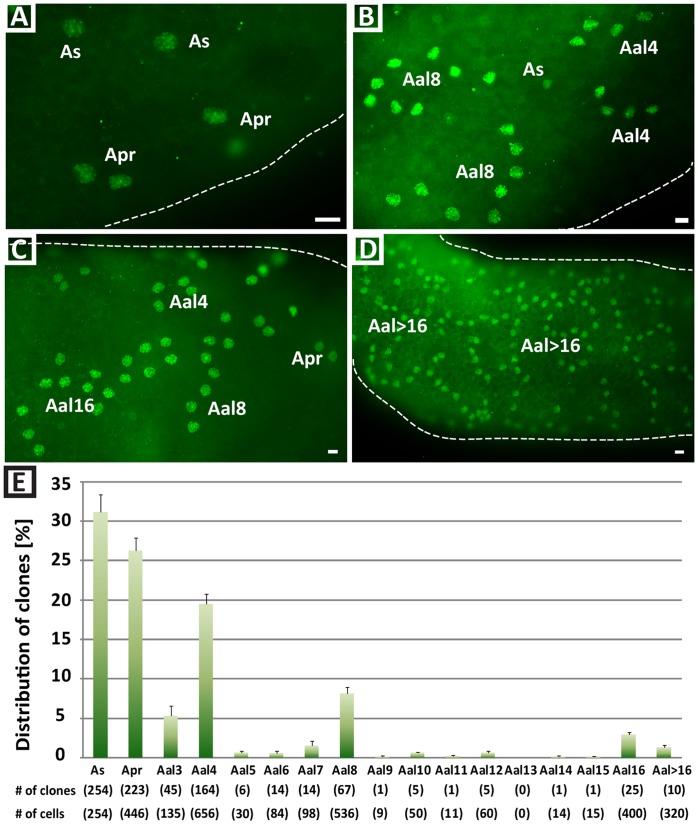
Clonal organization of SALL4 expressing cells in adult mouse seminiferous tubules. Whole mount seminiferous tubules were isolated from adult animals and subjected to immunofluorescence staining. (A–D) SALL4 was found to be expressed in A_s_, A_pr_ and A_al_ spermatogonia. Scale bars = 10 µm. (E) Quantification of the frequency of SALL4 positive A_s_, A_pr_ and A_al_ spermatogonial clones that were observed in adult mouse seminiferous tubules. Data are presented as average ± SEM from three independent experiments. The number of clones counted is indicated in parentheses beneath the graph. Total cell numbers in each clone size were calculated by multiplying the number of clones by the number of cells contained in each clone of a certain size, e.g. 254 A_s_ clones = 254 cells, 223 A_pr_ clones = 446 cells, etc. Cell numbers are shown in parentheses.

To correlate SALL4 expression with spermatogonial clone size (A_s,_ A_pr_, A_al_), we performed SALL4 immunohistochemistry in whole mount preparations of seminiferous tubules (Fig. 5A–D). We found that SALL4 positive cells were arranged as A_singles_ (31.4%±2.2 of SALL4 positive clones), A_pairs_ (26.3±1.6%), chains of A_al4_ (19.5±1.3%), A_al8_ (8.1±0.9%), A_al16_ (2.9±0.3%) and sometimes in longer chains of A_al>16_ spermatogonia (1.3±0.3%) (Fig. 5E). Clones of 3 SALL4^+^ cells were occasionally observed. Otherwise odd clone sizes that did not correspond to population doubling divisions (2, 4, 8, 16) were rarely observed.

### SALL4 is Co-expressed with PLZF and LIN28 in A_s_, A_pr_ and A_al_ Spermatogonia

**Figure 6:**
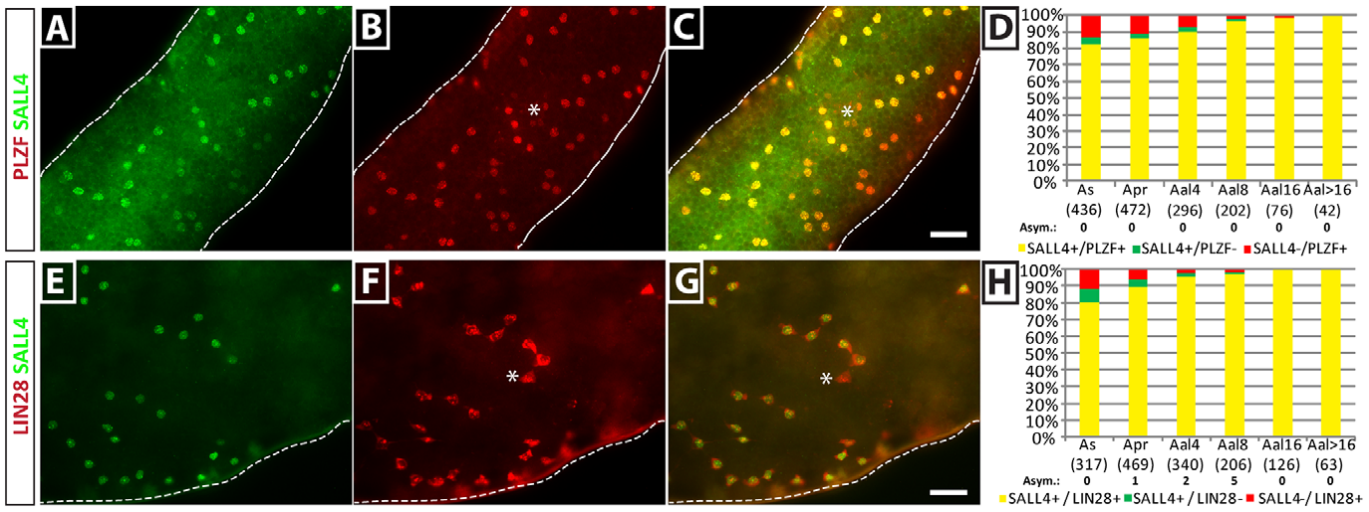
SALL4 is co-expressed with PLZF and LIN28 in clones of undifferentiated spermatogonia. (A–D) SALL4 and PLZF were co-expressed in most undifferentiated spermatogonia. Small fractions of A_s_ and A_pr_ spermatogonia showed molecular heterogeneity (*). (E–H) SALL4 also largely overlaps with LIN28 expression, with heterogeneity of expression observed in few A_s_ and A_pr_ spermatogonial clones (*). Scale bars = 50 µm. Data obtained from n = 3 animals, presented as average ± SEM. Asym: Number of clones with molecular asymmetry.

It was previously reported that the A_undiff_ population is heterogeneous [28], [29]. In order to determine the distribution of SALL4 in spermatogonial clones in relation to other known markers, we used whole mount immunohistochemical co-staining (Fig. 6). In accordance with the data from tissue section immunostaining, we found that SALL4 expression substantially overlapped with both PLZF and LIN28. In particular, PLZF was co-expressed with SALL4 in 82.7±2.2% of A_s_ spermatogonia, 86.3±0.7% of A_pr_ spermatogonia and 90.3±2.0% of A_al4_ spermatogonia. Co-expression further increased in chains of eight and 16 cells (96.6±1.7% and 98.7±1.3%, respectively) and was complete in all clones observed with more than 16 cells. SALL4 and LIN28 were co-expressed in 77.9±3.2% of A_s_ and 88.3±1.1% of A_pr_ spermatogonia. As with PLZF, co-expression of SALL4 and LIN28 increased in A_al4_ and A_al8_ (95.4±1.9% and 97.2±0.7%, respectively) and was complete in all chains containing 16 or more spermatogonia. Asymmetrical clones of A_pr_ and A_al_ spermatogonia, as previously reported by others [30], [31], [32], were observed infrequently (<1% of clones, see Fig. 6D and H) and were counted as double stained clones because it is not possible to know whether the asymmetry is biologically relevant or an artifact caused by tubule preparation or staining procedure.

Thus, it appears that most A_undiff_ express SALL4, but some minor populations of SALL4-only, PLZF-only and LIN28-only expressing cells were present in the seminiferous epithelium. In this regard it is interesting to note that heterogeneity of expression was greater in A_s_ and A_pr_ spermatogonia than in A_al_ spermatogonia. The biological significance of these subpopulations for spermatogonial self-renewal and differentiation remains unclear, but this observation is consistent to reports by others and will require further investigation [29], [30], [33].

### Differential Expression of GFRα1 and SALL4 in A_s_ and A_pr_ Spermatogonia

The progression of spermatogonia from A_s_ to A_al_ is thought to be indicative of a cell fate shift towards differentiation and thus, the spermatogonial stem cell potential to self-renew is primarily attributed to A_s_ and sometimes A_pr_ spermatogonia. We examined this phenomenon further by evaluating the co-expression of SALL4 with GFRα1, one of the most restricted markers for undifferentiated spermatogonia [3].

**Figure 7:**
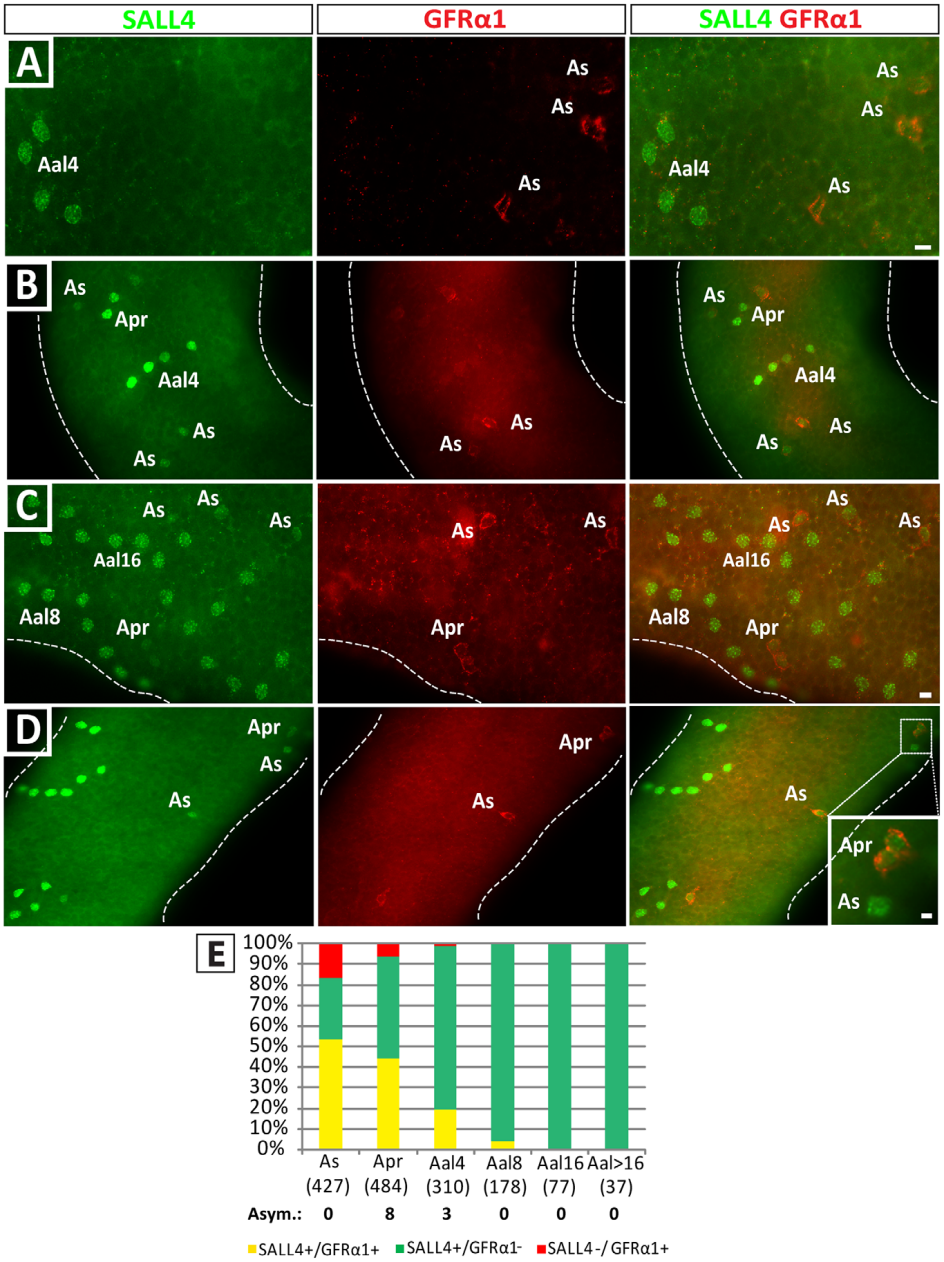
Differential expression of GFRα1 and SALL4 in A_s_ and A_pr_ spermatogonia. Expression of GFRα1 was limited to short chains of A_undiff_ and more restricted than expression of SALL4. (A–D) Examples of GFRα1 and SALL4 expressing spermatogonial clones in whole mount seminiferous tubules. Scale bars = 10 µm. Panel D was modified from [1] with permission. (E) Quantitative evaluation of GFRα1 and SALL4 expression in clones of undifferentiated spermatogonia. Among A_s_ spermatogonia, 50% of cells co-expressed both markers, but substantial populations of SALL4-only and GFRα1-only cells were also observed. The number of SALL4-only cells increased in longer chains, when GFRα1 expression ceased. Asym: Number of clones with molecular asymmetry.

Glial cell line-derived neurotrophic factor (GDNF) is essential for SSC maintenance and self-renewal both in vitro and in vivo [34], [35] and its receptor GFRα1 is expressed on a subset of undifferentiated spermatogonia [30]. Here, we used double-immunofluorescence to investigate if SALL4 is present in the same spermatogonial population as this putative SSC marker (Fig. 7). As expected, GFRα1 expression was restricted primarily to A_s_, A_pr_ and some A_al4_ spermatogonia. It was rarely observed in A_al8,_ and was never observed in chains larger than 8.

Substantial heterogeneity between SALL4 and GFRα1 was observed in A_s_ and A_pr_ spermatogonia. In particular, 51.7±7.8% of A_s_ co-expressed both markers, but 30.8±7.4% of A_s_ were SALL4 positive only and the remaining 17.6±3.9% A_s_ spermatogonia expressed only GFRα1. Similarly, 44.3±7.2% of the A_pr_ spermatogonia co-expressed SALL4 and GFRα1, 49.7±7.0% expressed SALL4 only and 6±0.3% expressed GFRα1 only. In A_al4_, only 19.6±2.9% of the cells were SALL4+/GFRα1+ and the dominant phenotype was SALL4+ only (79.2±3.0%). GFRα1/SALL4 co-expression was observed in only 4% of A_al8_ and GFRα1 expression was not observed in chains of 16 or more aligned spermatogonia, resulting in a singular SALL4+ phenotype. In rare events, asymmetrical A_pr_ and A_al4_ were observed (<1%, see Fig. 7).

### SALL4 is Downregulated in cKIT-positive, Differentiating Spermatogonia

**Figure 8:**
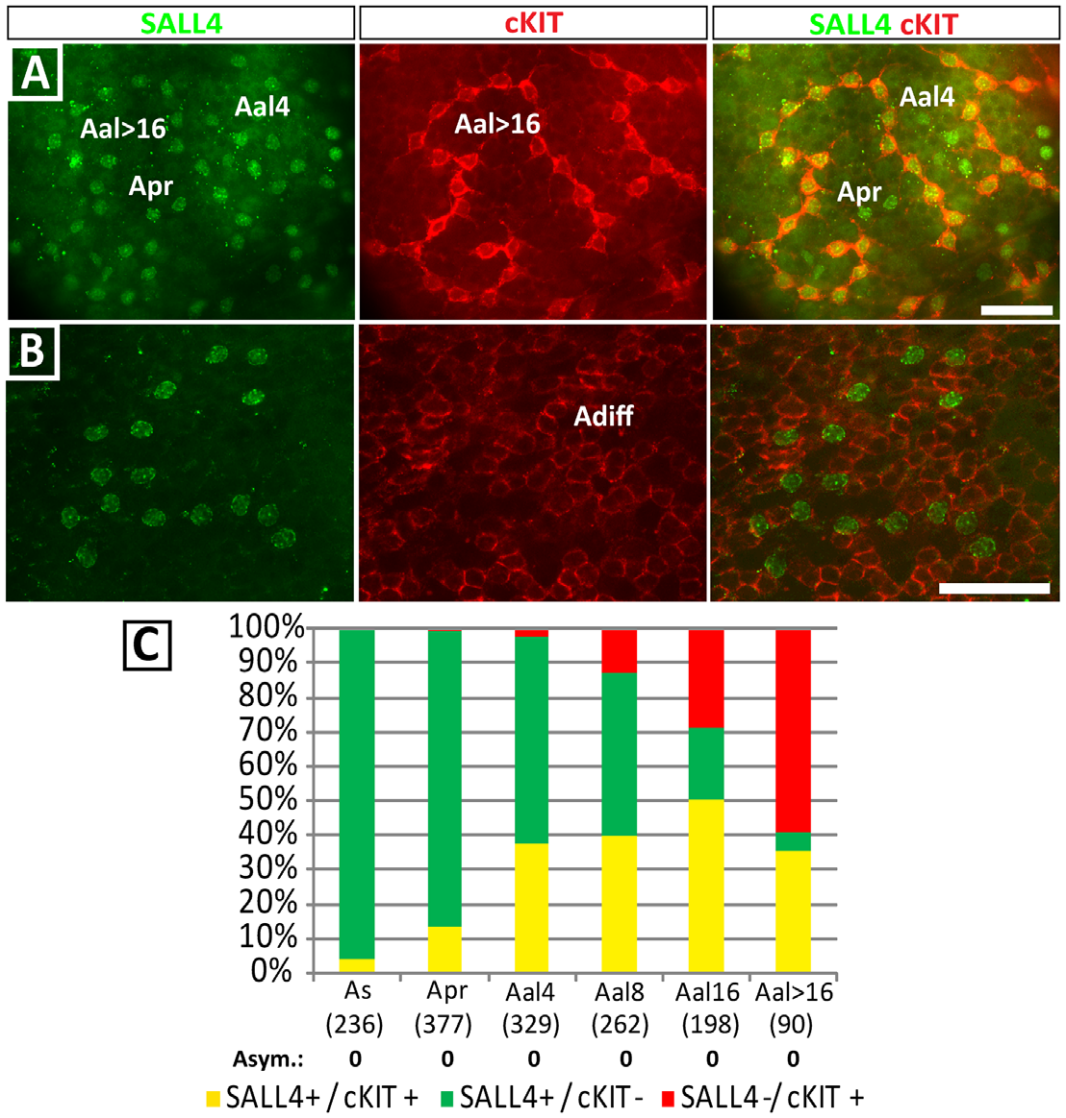
SALL4 is downregulated in differentiating spermatogonia. (A) SALL4 and cKIT are co-expressed in chained A_al_ spermatogonia. (B) SALL4 becomes downregulated as spermatogonia differentiate. In contrast, cKIT expression persists in differentiated germ cells. (C) Quantification of SALL4/cKIT co-staining showed that SALL4 expression decreased in larger clones coincident with increased cKIT expression. Note that cKIT expression in A_al16_ remained heterogeneous. Scale bars = 50 µm. Asym: Number of clones with molecular asymmetry.

Next, we investigated the expression of SALL4 in spermatogonia poised to undergo differentiation. The cKIT receptor tyrosine kinase is upregulated during the transition of undifferentiated spermatogonia into differentiating A1 spermatogonia and remains expressed during the amplifying pre-meiotic spermatogonial divisions [36]. cKIT expression (SALL4+/cKIT+ and SALL4−/cKIT+) was occasionally observed in the smallest A_s_ (4.1%) and A_pr_ (14.3%) clones, and expression increased markedly in A_al4_, when 39.8% of spermatogonia expressed cKIT (Fig. 8). However, at this point the majority of A_al4_ (60.3±8.7%) were SALL4+ only. At the stage of A_al8_, cKIT expression became more prominent, with increasing numbers of SALL4+/cKIT+ clones (40.7%) and cKIT+ only clones (12.3%) being observed. Most A_al16_ spermatogonia expressed cKIT (79.0%), but it is important to note that a substantial number of A_al16_ were SALL4+/cKIT- (21.0±5.14%) (Fig. 8B). Taken together, these results indicate that SALL4 expression declines in larger A_undiff_ clones coincident with increasing cKIT expression. Asymmetrical clones were not observed.

**Figure 9:**
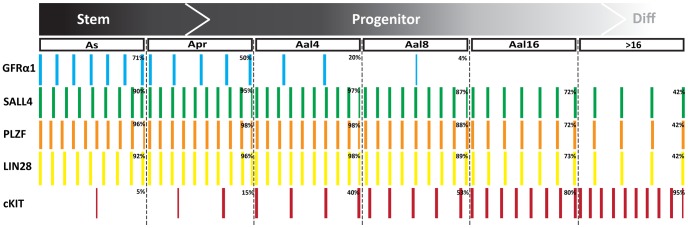
Summary of spermatogonial marker expression in mouse spermatogonia. Graphical summary of quantitative data from Figures 6–8 were used to calculate the ratio of cells expressing each marker within the populations of A_s_, A_pr_, and A_al_ spermatogonia. Each bar represents 10% GFRα1-, SALL4-, PLZF-, LIN28- or cKIT- positive cells, respectively, and half bars represent approximately 5% positive cells. For example, co-staining revealed that 71% of A_s_ spermatogonia expressed GFRα1, therefore 7 bars (each representing 10%), are shown in the diagram. The heterogeneity of the molecular phenotype of spermatogonia clone sizes is represented by the extent of overlap among bars across marker profiles.

The expression data from all whole mount immunostaining experiments are summarized in Figure 9.

## Discussion

SALL4 participates in transcriptional regulatory networks during fetal development of various organs [21] and is critical for cell fate decisions and lineage specification [16]. In contrast to most organs, expression in the germline is maintained after birth [25] and is restricted to pre-meiotic germ cells in rodents, non-human primates and humans [23], [26]. Although the exact function of SALL4 in spermatogonia is still unclear, increasing knowledge from studies of embryonic stem cells and hematopoietic stem cells may provide clues about possible mechanisms of SALL4 action in germ cells [37], [38], [39], [40]. Here, we analyzed the expression pattern of SALL4 during spermatogenic lineage development and steady-state spermatogenesis in relation to clone size and established markers for undifferentiated and differentiating spermatogonia.

Our results show that SALL4 is expressed by a subpopulation of quiescent gonocytes at PND0, but is expressed by all gonocytes at PND3, which coincides with re-entry into the cell cycle and migration to the seminiferous tubule basement membrane [5]. A similar pattern has been reported for neonatal and postnatal germ cells in the marmoset [26]. The pool of perinatal gonocytes has two destinies; differentiate to produce the first round of spermatogenesis and establish the initial pool of spermatogonial stem cells [6], [41]. Perhaps the heterogeneous SALL4 expression at PND0 distinguishes these two populations. However, by day 7 in our study, when gonocytes reach the basement membrane, SALL4 was expressed in all germ cells. Moreover, the observation that two SALL4 splice variants are expressed differentially during this specific time when the SSC pool is established may suggest that the SALL4A and SALL4B isoforms possess different regulatory functions in germ cells. To our knowledge, this is the first study that identified differential expression of SALL4 isoforms in undifferentiated spermatogonia. It was previously shown that SALL4A and SALL4B can form homo- or heterodimers with distinct DNA target regions regulating either pluripotency genes (SALL4A/B heterodimers, SALL4B/B homodimers) or differentiating/patterning genes (SALL4A/A homodimers) [27]. Thus, the presence of SALL4B alone (in gonocytes), or SALL4A and SALL4B (in PND7 spermatogonia) may have implications for the regulation of target genes, perhaps influencing the fate toward stem cell or differentiation to produce the first round of spermatogenesis. However, we were not able to identify subpopulations of spermatogonia at PND7 or 14 with the phenotypes SALL4A only or SALL4B only; rather, all spermatogonia had the SALL4A/B co-stained phenotype. It is possible that the ratio of SALL4A to SALL4B changes on a per cell basis, but this could not be unequivocally confirmed by immunohistochemistry.

We then investigated SALL4 expression during steady-state spermatogenesis. Our data confirmed previous reports of SALL4 co-localization with PLZF, an established marker for undifferentiated spermatogonia, in tissue sections [31]. We further show that SALL4 is almost completely co-expressed with a second spermatogonia marker, the RNA-binding protein LIN28. In contrast, GATA4-expressing Sertoli cells did not express SALL4. We observed 16.6 Sertoli cells per tubular cross section, which is comparable to data reported by Oatley and coworkers [42], and using this approach we calculated that approximately 800 SALL4-positive spermatogonia per 1000 Sertoli cells are present in the adult mouse testis. It is important to note that the size of the SALL4-positive spermatogonial population was similar to the population of PLZF-positive and LIN28-positive spermatogonia. Because of the extensive overlap between SALL4, PLZF, and LIN28 expression, and almost identical population size relative to Sertoli cell number, we propose that SALL4 is expressed in undifferentiated spermatogonia.

Studies in tissue sections do not provide information about the topographical arrangement of spermatogonial clones (A_s_, A_pr_, A_al_), which might be related to stage of differentiation. It is therefore important to correlate the molecular phenotype of spermatogonia with their clonal organization by whole-mount seminiferous tubule immunofluorescence staining. Using this approach, we observed SALL4 in A_s_, A_pr_ and A_al4,8,16_ spermatogonia. Occasionally, clones with an odd number of cells (A_al3_, A_al6_, A_al12_) were observed. These could be the result of clone fragmentation, a phenomenon that has been described by Nakagawa et al. [3] and that was proposed as a mechanism to replenish the spermatogonial pool. Further, extensive co-localization studies in whole-mount samples revealed significant co-expression of SALL4 with PLZF and LIN28 in the majority of A_undiff_ spermatogonia.

Germ cell differentiation is regulated by endocrine and paracrine factors that signal either to the somatic component of the seminiferous tubules, the Sertoli cells, or directly to spermatogonial stem cells to elicit self-renewing or differentiation divisions. A key factor for SSC survival and renewal both in vivo and in vitro is glial cell line-derived neurotrophic factor (GDNF) [34], [35] that is secreted by Sertoli cells [43] and binds to the RET/GFRα1 receptor complex [44] on undifferentiated spermatogonia including SSCs [45]. Accordingly, the presence of GFRα1 on the cell surface of putative SSCs is well characterized and was found to be restricted predominantly to A_s_, A_pr_ and A_al4_ [30]. In contrast to the PLZF and LIN28 results, we observed a high degree of heterogeneity among A_s_ spermatogonia in SALL4 and GFRα1 co-staining experiments. Grisanti et al. reported that 10% of the PLZF-expressing A_s_ spermatogonia are GFRα1-negative and that the stem cell activity in this population is greater than in GFRα1 positive cells, as determined by transplantation assay [30]. In the study mentioned above, Grisanti et al. also observed that ∼5% of PLZF-positive A_pr_ spermatogonia had asymmetrical GFRα1 expression. In our study, we rarely observed asymmetrical clones (either SALL4 asymmetric or GFRα1 or LIN28 asymmetric, <1% of clones). The reason that we observed slightly fewer asymmetric clones than previously reported could be that Grisanti et al. made counts from images, while we counted clones directly on the microscope, which allowed us to follow clones through different focal planes.

Here, we found that only half of the A_s_ population co-expressed GFRα1 and SALL4, while the other half consisted of SALL4 only or GFRα1 only cells. The biological function and the actual stem cell potential in these different spermatogonial populations is not known and will have to be investigated in future studies, perhaps using transgenic tools.

We next performed co-staining experiments for SALL4 and the cKIT receptor tyrosine kinase, which is a marker for differentiating germ cells. cKit becomes upregulated as A_al_ transition to A1 spermatogonia, possibly through de-repression of cKit gene expression in the absence of PLZF [46]. In this study, we observed cKIT expression in a minor fraction of A_s_ and A_pr_, which increased in larger clones and was associated with a corresponding decrease in SALL4 expression in larger clones. Interestingly, a very small proportion of A_pr_ and A_al4_ clones expressed cKIT only (without SALL4), suggesting that the A_al_ to A1 transition might sometimes occur from small clones, as previously described in the Chinese hamster [47]. While the majority of A_al16_ clones expressed cKIT (either with or without SALL4), we did observe A_al16_ SALL4+ clones that were cKIT negative.

Hobbs et al. recently demonstrated a direct interaction of SALL4 and PLZF in transfected HEK293 cells and reported that the two factors can displace each other from their cognate chromatin locations depending on their relative protein levels [23]. PLZF is a repressor of the differentiation factor, cKIT, and the authors proposed that SALL4 might act as an important spermatogonial differentiation factor that functions by removing PLZF from its cognate targets (e.g. cKIT). Does this suggest that the SALL4+/PLZF- As and Apr spermatogonia express cKIT and are destined to differentiate? In this regard, triple staining experiments would be very informative, but are difficult to execute due to availability of compatible antibodies and differences in optimal staining conditions for each marker.

Taken together, our data from cross-section and whole mount preparations of seminiferous tubules corroborate earlier reports of molecular heterogeneity among A_undiff_ spermatogonia, which may indicate functional heterogeneity among clone sizes [29], [30], [32], [33]. The heterogeneity can only be observed when co-staining with more than one marker is performed, therefore caution is required for the interpretation of experiments that use only a single marker as a denominator for A_undiff_ spermatogonia. In this study, we report that SALL4 is a molecular marker for undifferentiated A_s_, A_pr_ and A_al_ spermatogonia, a population that includes spermatogonial stem cells, and that it can be used in combination with other molecular markers to dissect spermatogonial lineage development and steady state spermatogenesis in the postnatal testis.

## Supporting Information

Figure S1 **Immunostaining of tissue sections with anti-SALL4 and anti-DAZL IgG.** Fluorescent signal was specific for SALL4 and DAZL (A) and no fluorescent staining signal was observed in Isotype controls (B). Scale bar = 10 µm.
(TIF)

Figure S2 **Immunostaining of adult mouse testis sections with spermatogonial markers.** Fluorescent signal was specific for SALL4, PLZF and LIN28 (A, C) and no fluorescent staining signal was observed in Isotype controls (B, D). Scale bar = 10 µm.
(TIF)

Figure S3 **Immunostaining and controls of whole mount seminiferous tubules.** Fluorescent signal was specific for SALL4, PLZF, LIN28, GFRα1, or cKIT (left column) and no fluorescent staining signal was observed in Isotype controls (right column). Scale bar = 50 µm.
(TIF)

## References

[1] Phillips BT, Gassei K, Orwig KE (2010) Spermatogonial stem cell regulation and spermatogenesis. Philos Trans R Soc Lond B Biol Sci 365: 1663–1678. 20403877 10.1098/rstb.2010.0026 PMC2871929

[2] de Rooij DG, Russell LD (2000) All you wanted to know about spermatogonia but were afraid to ask. J Androl 21: 776–798. 11105904

[3] Nakagawa T, Sharma M, Nabeshima Y, Braun RE, Yoshida S (2010) Functional hierarchy and reversibility within the murine spermatogenic stem cell compartment. Science 328: 62–67. 20299552 10.1126/science.1182868 PMC2981100

[4] Tegelenbosch RA, de Rooij DG (1993) A quantitative study of spermatogonial multiplication and stem cell renewal in the C3H/101 F1 hybrid mouse. Mutat Res 290: 193–200. 7694110 10.1016/0027-5107(93)90159-d

[5] Culty M (2009) Gonocytes, the forgotten cells of the germ cell lineage. Birth Defects Res C Embryo Today 87: 1–26. 19306346 10.1002/bdrc.20142

[6] Yoshida S, Sukeno M, Nakagawa T, Ohbo K, Nagamatsu G, et al (2006) The first round of mouse spermatogenesis is a distinctive program that lacks the self-renewing spermatogonia stage. Development 133: 1495–1505. 16540512 10.1242/dev.02316

[7] Jürgens G (1988) Head and tail development of the Drosophila embryo involves spalt, a novel homeotic gene. EMBO J 7: 189–196. 16453820 10.1002/j.1460-2075.1988.tb02799.x PMC454247

[8] Kühnlein RP, Frommer G, Friedrich M, Gonzalez-Gaitan M, Weber A, et al (1994) spalt encodes an evolutionarily conserved zinc finger protein of novel structure which provides homeotic gene function in the head and tail region of the Drosophila embryo. EMBO J 13: 168–179. 7905822 10.1002/j.1460-2075.1994.tb06246.x PMC394790

[9] Hollemann T, Schuh R, Pieler T, Stick R (1996) Xenopus Xsal-1, a vertebrate homolog of the region specific homeotic gene spalt of Drosophila. Mech Dev 55: 19–32. 8734496 10.1016/0925-4773(95)00485-8

[10] Camp E, Hope R, Kortschak RD, Cox TC, Lardelli M (2003) Expression of three spalt (sal) gene homologues in zebrafish embryos. Dev Genes Evol 213: 35–43. 12590351 10.1007/s00427-002-0284-6

[11] Sweetman D, Smith T, Farrell ER, Chantry A, Munsterberg A (2003) The conserved glutamine-rich region of chick csal1 and csal3 mediates protein interactions with other spalt family members. Implications for Townes-Brocks syndrome. J Biol Chem 278: 6560–6566. 12482848 10.1074/jbc.M209066200

[12] Ott T, Kaestner KH, Monaghan AP, Schutz G (1996) The mouse homolog of the region specific homeotic gene spalt of Drosophila is expressed in the developing nervous system and in mesoderm-derived structures. Mech Dev 56: 117–128. 8798152 10.1016/0925-4773(96)00516-3

[13] Kohlhase J, Schuh R, Dowe G, Kuhnlein RP, Jackle H, et al (1996) Isolation, characterization, and organ-specific expression of two novel human zinc finger genes related to the Drosophila gene spalt. Genomics 38: 291–298. 8975705 10.1006/geno.1996.0631

[14] Sweetman D, Munsterberg A (2006) The vertebrate spalt genes in development and disease. Dev Biol 293: 285–293. 16545361 10.1016/j.ydbio.2006.02.009

[15] Uez N, Lickert H, Kohlhase J, de Angelis MH, Kuhn R, et al (2008) Sall4 isoforms act during proximal-distal and anterior-posterior axis formation in the mouse embryo. Genesis 46: 463–477. 18781635 10.1002/dvg.20421

[16] Elling U, Klasen C, Eisenberger T, Anlag K, Treier M (2006) Murine inner cell mass-derived lineages depend on Sall4 function. Proc Natl Acad Sci U S A 103: 16319–16324. 17060609 10.1073/pnas.0607884103 PMC1637580

[17] Oikawa T, Kamiya A, Kakinuma S, Zeniya M, Nishinakamura R, et al (2009) Sall4 regulates cell fate decision in fetal hepatic stem/progenitor cells. Gastroenterology 136: 1000–1011. 19185577 10.1053/j.gastro.2008.11.018

[18] Kohlhase J, Heinrich M, Schubert L, Liebers M, Kispert A, et al (2002) Okihiro syndrome is caused by SALL4 mutations. Hum Mol Genet 11: 2979–2987. 12393809 10.1093/hmg/11.23.2979

[19] Kohlhase J, Heinrich M, Liebers M, Frohlich Archangelo L, Reardon W, et al (2002) Cloning and expression analysis of SALL4, the murine homologue of the gene mutated in Okihiro syndrome. Cytogenet Genome Res 98: 274–277. 12826753 10.1159/000071048

[20] Warren M, Wang W, Spiden S, Chen-Murchie D, Tannahill D, et al (2007) A Sall4 mutant mouse model useful for studying the role of Sall4 in early embryonic development and organogenesis. Genesis 45: 51–58. 17216607 10.1002/dvg.20264 PMC2593393

[21] Sakaki-Yumoto M, Kobayashi C, Sato A, Fujimura S, Matsumoto Y, et al (2006) The murine homolog of SALL4, a causative gene in Okihiro syndrome, is essential for embryonic stem cell proliferation, and cooperates with Sall1 in anorectal, heart, brain and kidney development. Development 133: 3005–3013. 16790473 10.1242/dev.02457

[22] Koshiba-Takeuchi K, Takeuchi JK, Arruda EP, Kathiriya IS, Mo R, et al (2006) Cooperative and antagonistic interactions between Sall4 and Tbx5 pattern the mouse limb and heart. Nat Genet 38: 175–183. 16380715 10.1038/ng1707

[23] Hobbs RM, Fagoonee S, Papa A, Webster K, Altruda F, et al (2012) Functional antagonism between Sall4 and Plzf defines germline progenitors. Cell Stem Cell 10: 284–298. 22385656 10.1016/j.stem.2012.02.004 PMC3299297

[24] Yamatani H, Sato Y, Fujisawa H, Hirata T (2004) Chronotopic organization of olfactory bulb axons in the lateral olfactory tract. J Comp Neurol 475: 247–260. 15211465 10.1002/cne.20155

[25] Wang PJ, McCarrey JR, Yang F, Page DC (2001) An abundance of X-linked genes expressed in spermatogonia. Nat Genet 27: 422–426. 11279525 10.1038/86927

[26] Eildermann K, Aeckerle N, Debowski K, Godmann M, Christiansen H, et al (2012) Developmental Expression of the Pluripotency Factor Sal-Like Protein 4 in the Monkey, Human and Mouse Testis: Restriction to Premeiotic Germ Cells. Cells Tissues Organs 196: 206–220. 22572102 10.1159/000335031

[27] Rao S, Zhen S, Roumiantsev S, McDonald LT, Yuan GC, et al (2010) Differential roles of Sall4 isoforms in embryonic stem cell pluripotency. Mol Cell Biol 30: 5364–5380. 20837710 10.1128/MCB.00419-10 PMC2976381

[28] Sada A, Suzuki A, Suzuki H, Saga Y (2009) The RNA-binding protein NANOS2 is required to maintain murine spermatogonial stem cells. Science 325: 1394–1398. 19745153 10.1126/science.1172645

[29] Yoshida S, Nabeshima Y, Nakagawa T (2007) Stem cell heterogeneity: actual and potential stem cell compartments in mouse spermatogenesis. Ann N Y Acad Sci 1120: 47–58. 17905929 10.1196/annals.1411.003

[30] Grisanti L, Falciatori I, Grasso M, Dovere L, Fera S, et al.. (2009) Identification of Spermatogonial Stem Cell Subsets by Morphological Analysis and Prospective Isolation. Stem Cells: 3043–3052. 10.1002/stem.206 19711452

[31] Zheng K, Wu X, Kaestner KH, Wang PJ (2009) The pluripotency factor LIN28 marks undifferentiated spermatogonia in mouse. BMC Dev Biol 9: 38. 19563657 10.1186/1471-213X-9-38 PMC2719617

[32] Tokuda M, Kadokawa Y, Kurahashi H, Marunouchi T (2007) CDH1 is a specific marker for undifferentiated spermatogonia in mouse testes. Biol Reprod 76: 130–141. 17035642 10.1095/biolreprod.106.053181

[33] Suzuki H, Sada A, Yoshida S, Saga Y (2009) The heterogeneity of spermatogonia is revealed by their topology and expression of marker proteins including the germ cell-specific proteins Nanos2 and Nanos3. Dev Biol 336: 222–231. 19818747 10.1016/j.ydbio.2009.10.002

[34] Meng X, Lindahl M, Hyvonen ME, Parvinen M, de Rooij DG, et al (2000) Regulation of cell fate decision of undifferentiated spermatogonia by GDNF. Science 287: 1489–1493. 10688798 10.1126/science.287.5457.1489

[35] Kubota H, Avarbock MR, Brinster RL (2004) Culture conditions and single growth factors affect fate determination of mouse spermatogonial stem cells. Biol Reprod 71: 722–731. 15115718 10.1095/biolreprod.104.029207

[36] Yoshinaga K, Nishikawa S, Ogawa M, Hayashi S, Kunisada T, et al (1991) Role of c-kit in mouse spermatogenesis: identification of spermatogonia as a specific site of c-kit expression and function. Development 113: 689–699. 1723681 10.1242/dev.113.2.689

[37] Yuri S, Fujimura S, Nimura K, Takeda N, Toyooka Y, et al (2009) Sall4 is essential for stabilization, but not for pluripotency, of embryonic stem cells by repressing aberrant trophectoderm gene expression. Stem Cells 27: 796–805. 19350679 10.1002/stem.14

[38] Yang J, Aguila JR, Alipio Z, Lai R, Fink LM, et al (2011) Enhanced self-renewal of hematopoietic stem/progenitor cells mediated by the stem cell gene Sall4. J Hematol Oncol 4: 38. 21943195 10.1186/1756-8722-4-38 PMC3184628

[39] Yang J, Gao C, Chai L, Ma Y (2010) A novel SALL4/OCT4 transcriptional feedback network for pluripotency of embryonic stem cells. PLoS One 5: e10766. 20505821 10.1371/journal.pone.0010766 PMC2874005

[40] van den Berg DL, Snoek T, Mullin NP, Yates A, Bezstarosti K, et al (2010) An Oct4-centered protein interaction network in embryonic stem cells. Cell Stem Cell 6: 369–381. 20362541 10.1016/j.stem.2010.02.014 PMC2860243

[41] Kluin PM, de Rooij DG (1981) A comparison between the morphology and cell kinetics of gonocytes and adult type undifferentiated spermatogonia in the mouse. Int J Androl 4: 475–493. 7298230 10.1111/j.1365-2605.1981.tb00732.x

[42] Oatley MJ, Racicot KE, Oatley JM (2011) Sertoli cells dictate spermatogonial stem cell niches in the mouse testis. Biol Reprod 84: 639–645. 21084712 10.1095/biolreprod.110.087320 PMC3062034

[43] Hofmann MC (2008) Gdnf signaling pathways within the mammalian spermatogonial stem cell niche. Mol Cell Endocrinol 288: 95–103. 18485583 10.1016/j.mce.2008.04.012 PMC2491722

[44] Jing S, Wen D, Yu Y, Holst PL, Luo Y, et al (1996) GDNF-induced activation of the ret protein tyrosine kinase is mediated by GDNFR-alpha, a novel receptor for GDNF. Cell 85: 1113–1124. 8674117 10.1016/s0092-8674(00)81311-2

[45] Buageaw A, Sukhwani M, Ben-Yehudah A, Ehmcke J, Rawe VY, et al (2005) GDNF Family Receptor alpha1 Phenotype of Spermatogonial Stem Cells in immature Mouse Testes. Biology of Reproduction 73: 1011–1016. 16014811 10.1095/biolreprod.105.043810

[46] Filipponi D, Hobbs RM, Ottolenghi S, Rossi P, Jannini EA, et al (2007) Repression of kit expression by Plzf in germ cells. Mol Cell Biol 27: 6770–6781. 17664282 10.1128/MCB.00479-07 PMC2099235

[47] Lok D, Weenk D, De Rooij DG (1982) Morphology, proliferation, and differentiation of undifferentiated spermatogonia in the Chinese hamster and the ram. Anat Rec 203: 83–99. 7103128 10.1002/ar.1092030109

