# Supplementary figures and images for: SALL4 Expression in Gonocytes and Spermatogonial Clones of Postnatal Mouse Testes
Source: PLoS One. 2013 Jan 11;8(1):e53976. doi: 10.1371/journal.pone.0053976 (PMC3543410; doi:10.1371/journal.pone.0053976)

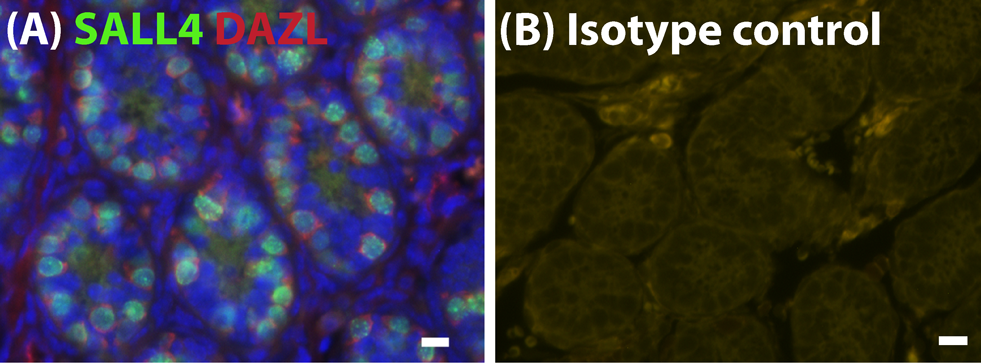

Supplement: Figure S1 — Immunostaining of tissue sections with anti-SALL4 and anti-DAZL IgG. Fluorescent signal was specific for SALL4 and DAZL (A) and no fluorescent staining signal was observed in Isotype controls (B). Scale bar = 10 µm. (TIF) [file pone.0053976.s001.tif]

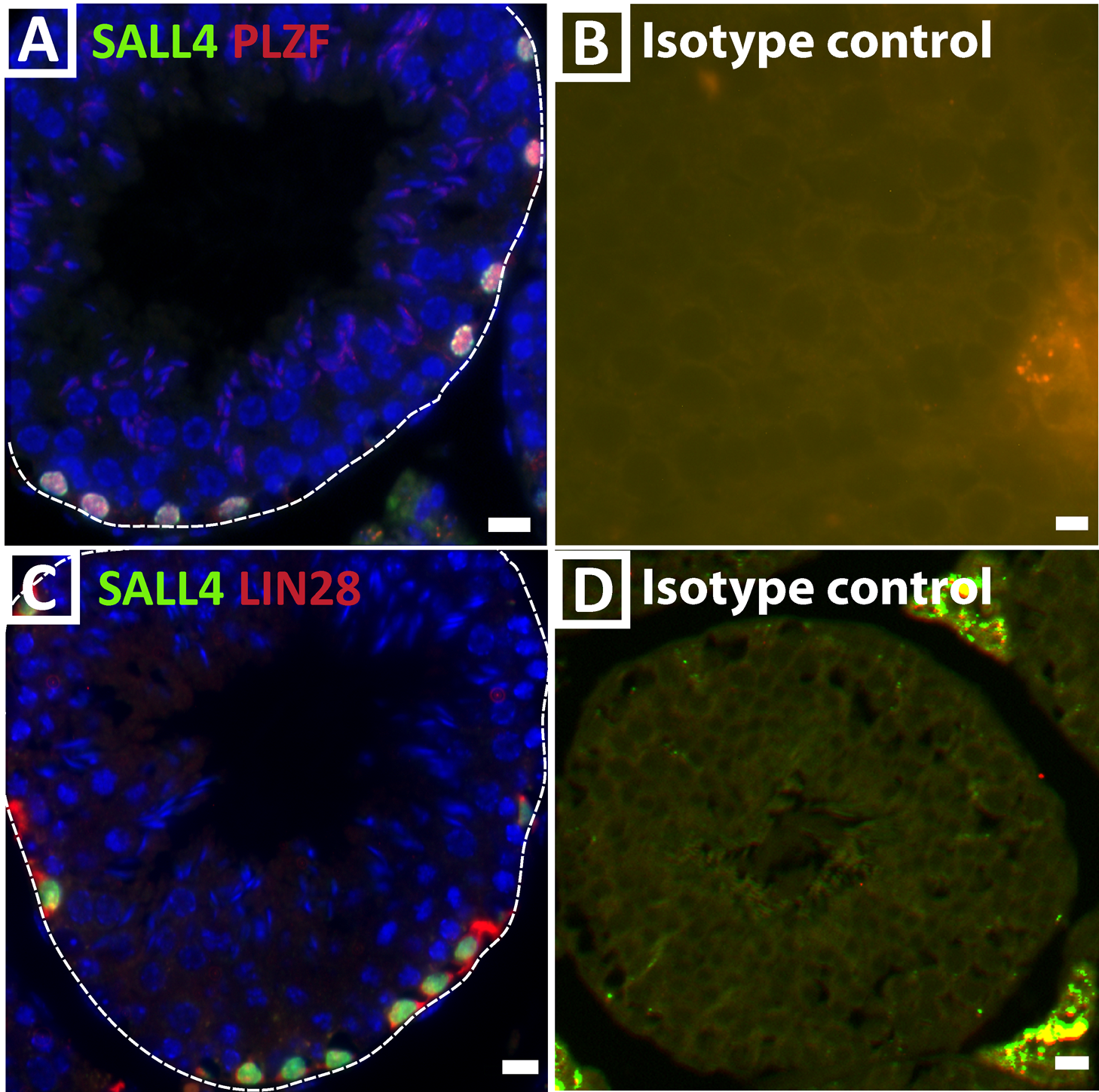

Supplement: Figure S2 — Immunostaining of adult mouse testis sections with spermatogonial markers. Fluorescent signal was specific for SALL4, PLZF and LIN28 (A, C) and no fluorescent staining signal was observed in Isotype controls (B, D). Scale bar = 10 µm. (TIF) [file pone.0053976.s002.tif]

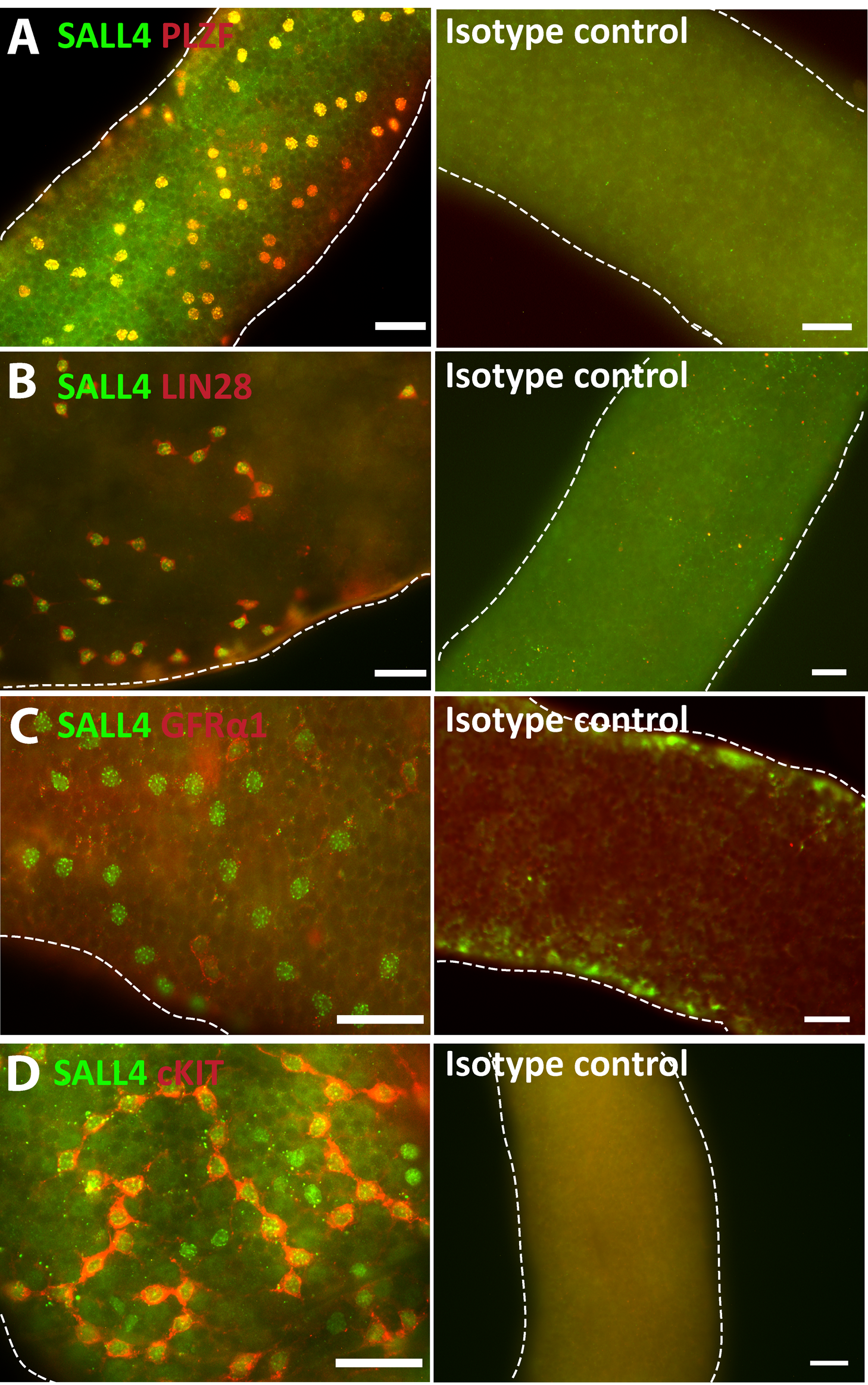

Supplement: Figure S3 — Immunostaining and controls of whole mount seminiferous tubules. Fluorescent signal was specific for SALL4, PLZF, LIN28, GFRα1, or cKIT (left column) and no fluorescent staining signal was observed in Isotype controls (right column). Scale bar = 50 µm. (TIF) [file pone.0053976.s003.tif]
